# Association of patterns of care, prognostic factors, and use of radiotherapy–temozolomide therapy with survival in patients with newly diagnosed glioblastoma: a French national population-based study

**DOI:** 10.1007/s11060-018-03065-z

**Published:** 2018-12-06

**Authors:** Pascale Fabbro-Peray, Sonia Zouaoui, Amélie Darlix, Michel Fabbro, Johan Pallud, Valérie Rigau, Hélène Mathieu-Daude, Faiza Bessaoud, Fabienne Bauchet, Adeline Riondel, Elodie Sorbets, Marie Charissoux, Aymeric Amelot, Emmanuel Mandonnet, Dominique Figarella-Branger, Hugues Duffau, Brigitte Tretarre, Luc Taillandier, Luc Bauchet

**Affiliations:** 1 Department of Biostatistics, Epidemiology, Public Health, CHU Nîmes, Nîmes, France; 2 EA2415 Research Unit, Montpellier University, Montpellier, France; 3 Department of Neurosurgery, Hopital Gui de Chauliac, CHU Montpellier, Montpellier University Medical Center, 80 Avenue Fliche, 34295 Montpellier, France; 4 INSERM U1051, Montpellier, France; 5 Groupe de Neuro-Oncologie du Languedoc Roussillon, ICM, Montpellier, France; 6 Department of Medical Oncology, ICM, Montpellier, France; 7 Department of Neurosurgery, Sainte Anne Hospital, and University Paris Descartes, Paris, France; 8 Department of Neuropathology, Hopital Gui de Chauliac, CHU Montpellier, Montpellier University Medical Center, Montpellier, France; 9 Department of Medical informatics, ICM, Montpellier, France; 10 Registre des Tumeurs de l’Hérault, ICM, Montpellier, France; 11 Department of Radiation Oncology, ICM, Montpellier, France; 12 Department of Neurosurgery, CHU Lariboisière, Paris, France; 13 Department of Neuropathology, CHU Marseille, INSERM U911, Marseille, France; 14 Department of Neuro-oncology, CHU Nancy, Nancy, France

**Keywords:** Clinical epidemiology, Glioblastoma, Neuro-oncology, Neurosurgery, Population-based study, Temozolomide

## Abstract

**Background:**

Glioblastoma is the most frequent primary malignant brain tumor. In daily practice and at whole country level, oncological care management for glioblastoma patients is not completely known.

**Objectives:**

To describe oncological patterns of care, prognostic factors, and survival for all patients in France with newly-diagnosed and histologically confirmed glioblastoma, and evaluate the impact of extended temozolomide use at the population level.

**Methods:**

Nationwide population-based cohort study including all patients with newly-diagnosed and histologically confirmed glioblastoma in France in 2008 and followed until 2015.

**Results:**

Data from 2053 glioblastoma patients were analyzed (male/female ratio 1.5, median age 64 years). Median overall survival (OS) was 11.2 [95% confidence interval (CI) 10.7–11.9] months. The first-line therapy and corresponding median survival (MS, in months) were: 13% did not receive any oncological treatment (biopsy only) (MS = 1.8, 95% CI 1.6–2.1), 27% received treatment without the combination of radiotherapy (RT)–temozolomide (MS = 5.9, 95% CI 5.5–6.6), 60% received treatment including the initiation of the concomitant phase of RT–temozolomide (MS = 16.4, 95% CI 15.2–17.4) whom 44% of patients initiated the temozolomide adjuvant phase (MS = 18.9, 95% CI 18.0–19.8). Only 22% patients received 6 cycles or more of adjuvant temozolomide (MS = 25.5, 95% CI 24.0–28.3). The multivariate analysis showed that the risk of mortality was significantly higher for the non-progressive patients who stopped at 6 cycles (standard protocol) than those who continued the treatment, hazard ratio = 1.5 (95% CI 1.2–1.9).

**Conclusion:**

In non-progressive patients, prolonging the adjuvant temozolomide beyond 6 cycles may improve OS.

**Electronic supplementary material:**

The online version of this article (10.1007/s11060-018-03065-z) contains supplementary material, which is available to authorized users.

## Introduction

Glioblastoma multiforme (GBM) is the most frequent malignant primitive brain tumor and the most deadly glioma subtype [1–3]. In daily practice, oncological care management for GBM patients is not completely standardized and depends on many factors [age, Karnofsky performance status (KPS), comorbidity, tumor (location, shape, and volume), etc.]. Few studies have directly led to improvements in medical care [4–10]. Since 2005, one pivotal clinical trial defined the radiotherapy and temozolomide combination (RT–temozolomide) as the standard of care in patients with newly-diagnosed GBM, for patients 18–70 years old with a WHO performance status of ≤ 2 [6]. Carmustine wafer (CW) is an option when maximal safe resection (RS) is performed and high grade glioma is histologically proven [11–14]. More recently, bevacizumab in combination with RT–temozolomide in first-line treatment showed a significant prolonged progression-free survival but failed to demonstrate a significant overall survival (OS) benefit [15, 16]. Moreover, “standards of care” do not exist for progression/recurrent GBM patients (e.g., bevacizumab was approved for treatment of recurrent GBM in USA, but not in Europe). One paper has shown the dissemination of RT–temozolomide combination as the treatment standard across countries, and a modest survival gain at the population level [17].

This work is requested by the French government and describes the oncological patterns of care, prognostic factors, and survival for all patients with newly-diagnosed and histologically confirmed GBM, for a country with more than 60 million inhabitants. This first paper aims to answer several questions concerning the first-line therapy at the national level: What surgery was performed (biopsy only, partial RS, total RS with or without CW implantation)? What extent of combined RT–temozolomide treatment did patients receive (concomitant phase only, initiation of adjuvant phase, 6 cycles of temozolomide in maintenance or more)? What was the survival for the different patient groups and did it differ between the group of patients with exactly 6 versus > 6 cycles of the adjuvant temozolomide treatment?

## Methods

The French Brain Tumor DataBase (FBTDB) identified and recorded all patients with newly-diagnosed and histologically confirmed primary brain tumors (e.g., GBM) since 2006 in France, and prospectively collected initial data. FBTDB is one of the largest clinical databases for brain tumors in Europe [18–26].

### Study population

This study includes all patients with newly-diagnosed and histologically confirmed GBM in 2008 (1 January–31 December). Histological diagnosis of GBM according to WHO 2007 classification [27] including glioblastoma, giant cell glioblastoma, and gliosarcoma, corresponding to ICD-O codes 9440/3, 9441/3, and 9442/3 respectively, were included. Exclusion criteria were previous surgery for GBM, spinal cord GBM, and patients from abroad or French overseas departments. A data card was used to retrospectively collect data on the oncological management [surgery, RT, and chemotherapy (CT)] and the follow-up care of these patients for the period 2008/01/01 to 2015/02/03. According to French neurosurgical guidelines, extent of RS should be evaluated with postoperative MRI. In our study, this information was reported from patient file. Central review was not performed.

The study was approved by the French legislation (CCTIRS n°10.548; CNIL n°911013).

### Treatment given

The start and end dates for RT and total dose were requested. For CT, the start and end dates and name and modality of administration were also requested. For the study of the RT–temozolomide treatment, the concomitant and the adjuvant phases were analyzed separately, and the number of cycles of adjuvant temozolomide was noted. Biopsy was considered a surgical procedure but not a treatment procedure.

### Statistical analysis

Statistical analysis was performed using SAS Enterprise Guide software, version 6.1. The analysis included a descriptive part of the original data and the oncological treatments received by the patients. Survival was estimated by the Kaplan–Meier method and defined as the time from first surgery (corresponding to the histological diagnosis) to death or censored at the date of last follow-up. The log-rank test was used to compare survival curves among different strata. The assumption of proportional hazards was verified before survival curve comparisons. Univariate Cox regression model was used to estimate hazard ratios (HRs) of strata versus reference level and their 95% Wald confidence interval (CI). For continuous variables, log linearity was tested before HR estimations. Multivariate Cox regression model was used to determine independent prognostic factors of OS. Potential prognostic factors and interaction terms were introduced into the model according to the univariate analysis (p-entry = 0.20), and a backward selection strategy was applied. We regarded p values < 0.05 as statistically significant. The Bonferroni correction was used for the multiple comparison tests. In order to assess duration of adjuvant phase of the RT–temozolomide combination as a prognostic factor, after 6 cycles of temozolomide without progression, patients with the treatment termination information as “end of treatment as defined by the Stupp protocol” and all the patients > 6 cycles, were selected and analyzed according to the same statistical strategy.

## Results

Of the 2167 cases identified by FBTDB, 114 were excluded (89 recurrences, 6 other histologic diagnoses, 4 duplicates and 15 patients from abroad). Therefore, this study included 2053 newly-diagnosed and histologically confirmed GBM patients. Of the 54 participating neurosurgical departments, 44 were public centers (36 university hospitals, 2 army hospitals, 4 general hospitals, and 2 non-profit institutions) and 10 were private institutions; the majority of patients (93.8%) were treated in public centers.

### Population characteristics

Median age at diagnosis was 64 years and more than a quarter of patients were > 72 years. Median preoperative KPS was 80% (range 20–100%). Preoperative KPS was available for only 916 patients (45%). Histological diagnosis was obtained during the initial surgery (RS 59%, biopsy 41%) (Table 1).

**Table 1:** Clinical characteristics of the 2053 patients at baseline

Characteristic (no. reported)	N	%
Sex (2053)		
Male	1232	60.0
Female	821	40.0
Age per quartile, in years (2053)		
≤ 56	515	25.1
57–63	460	22.4
64–72	547	26.6
> 72	531	25.9
Signs and symptoms (2046)		
Epilepsy	450	22.0
Headache	655	32.0
High intracranial pressure	285	13.9
Mental status disorders	930	45.4
Sensory-motor deficit	924	45.2
Other	352	17.2
Time between first sign and histological diagnosis, in months (1667)		
< 1	784	47.0
1–2	346	20.8
2–3	270	16.2
3–4	119	7.1
≥ 4	148	8.9
Preoperative KPS (916)		
90–100%	456	49.8
70–80%	338	36.9
≤ 60%	122	13.3
Location of the tumor (1869)		
Right	880	47.1
Left	855	45.7
Median and/or bilateral	134	7.2
Modality of the histological diagnosis (first surgery) (2053)		
“Total” RS	476	23.2
Partial RS	422	20.5
NOS RS	309	15.1
Biopsy	846	41.2
Histological diagnosis (2053)		
Glioblastoma	1988	96.8
Giant cell glioblastoma	36	1.8
Gliosarcoma	29	1.4

*KPS* Karnofsky performance status, *NOS* not otherwise specified, *RS* resection

### Overall survival and main spontaneous prognostic factors

Median OS was 11.2 (95% CI 10.7–11.9) months, [13.6 (95% CI 12.7–14.7) and 6.8 (95% CI 5.8–7.5) months for patients ≤ 70 and > 70 years old, respectively]. Survival rates at 1/1.5/2/ and 5 years were: 47.1% (95% CI 44.8–49.3%)/31.4% (95% CI 29.3–33.5%)/20.1% (95% CI 18.3–22.0%)/and 4.5% (95% CI 3.6–5.6%) (Supplementary Fig. S1). The main prognostic factors were age, tumor location, and KPS. Notably: the median survival (MS) of the group “KPS reported” versus “KPS unreported” was not significantly different (MS = 11.7 vs. 10.9 months, p = 0.50) (Supplementary Fig. S2).

### Oncological management in first-line therapy

Complete treatment information (including lack of treatment) was available for 1856 patients (Table 2). Nearly 60% of all patients initiated the concomitant phase of the combined RT–temozolomide treatment (group 1, n = 1111). In this group, RS was performed in 805 patients (72.5%), and 107 patients (9.6%) received CW implantation. Nearly 45% of all patients (n = 821) initiated the adjuvant phase, and only 21.6% of all patients (n = 401) received 6 cycles or more of temozolomide during the adjuvant phase. Among all patients, approximately 40% did not receive the combined RT–temozolomide treatment (group 2; n = 745) including 372 patients (20%) who did not receive any oncological treatment after surgery.

**Table 2:** First-line therapy (N = 1856)

First-line therapy^a,b^	N	%
Group 1: first-line including combined radiotherapy and temozolomide (RT–temozolomide) treatment	1111	59.9
Surgery + RT–concomitant temozolomide initiated	1111	
RS + RT–concomitant temozolomide initiated	805	
RS without CW	698	
RS with CW	107	
B + RT–concomitant temozolomide initiated	306	
Surgery + RT–concomitant temozolomide + adjuvant temozolomide initiated	821	44.2
RS + RT–concomitant temozolomide + adjuvant temozolomide initiated	638	
RS without CW	561	
RS with CW	77	
B + RT–concomitant temozolomide + adjuvant temozolomide initiated	183	
Surgery + RT–concomitant temozolomide + adjuvant temozolomide < 6 cycles	420	22.6
RS + RT–concomitant temozolomide + adjuvant temozolomide < 6 cycles	308	
RS without CW	275	
RS with CW	33	
B + RT–concomitant temozolomide + adjuvant temozolomide < 6 cycles	112	
Surgery + RT–concomitant temozolomide + adjuvant temozolomide = 6 cycles^c^	220	11.9
RS + RT–concomitant temozolomide + adjuvant temozolomide = 6 cycles	190	
RS without CW	162	
RS with CW	28	
B + RT–concomitant temozolomide + adjuvant temozolomide = 6 cycles	30	
Surgery + RT–concomitant temozolomide + adjuvant temozolomide > 6 cycles	181	9.8
RS + RT–concomitant temozolomide + adjuvant temozolomide > 6 cycles	140	
RS without CW	124	
RS with CW	16	
B + RT–concomitant temozolomide + adjuvant temozolomide > 6 cycles	41	
Group 2: first-line excluding combined RT–temozolomide treatment	745	40.1
B alone (no treatment)	241	13.0
RS alone (no other treatment)	131	7.1
B + CT	156	8.4
Temozolomide 5/28	118	
Bevacizumab	15	
Other CT	23	
B + RT	53	2.9
RS + CT	66	3.6
Temozolomide 5/28	38	
Bevacizumab	6	
Other CT	22	
RS + RT	70	3.8
Other	28	

*B* biopsy, *CT* chemotherapy, *CTconco* concomitant chemotherapy, *CW* carmustine wafer, *RS* resection, *RT* radiotherapy

^a^Median duration and median dose for RT were 44 days and 60 Gy respectively

^b^Median times from surgery to the next treatment with RT–temozolomide, CT alone, or RT alone were 39, 20, 40 days respectively

^c^Of the 220 patients who received exactly 6 cycles of adjuvant temozolomide, the treatment termination information for “end of treatment as defined by the Stupp protocol” was specified in 166 cases

### Survival according to the first-line therapy

**Fig. 1:**
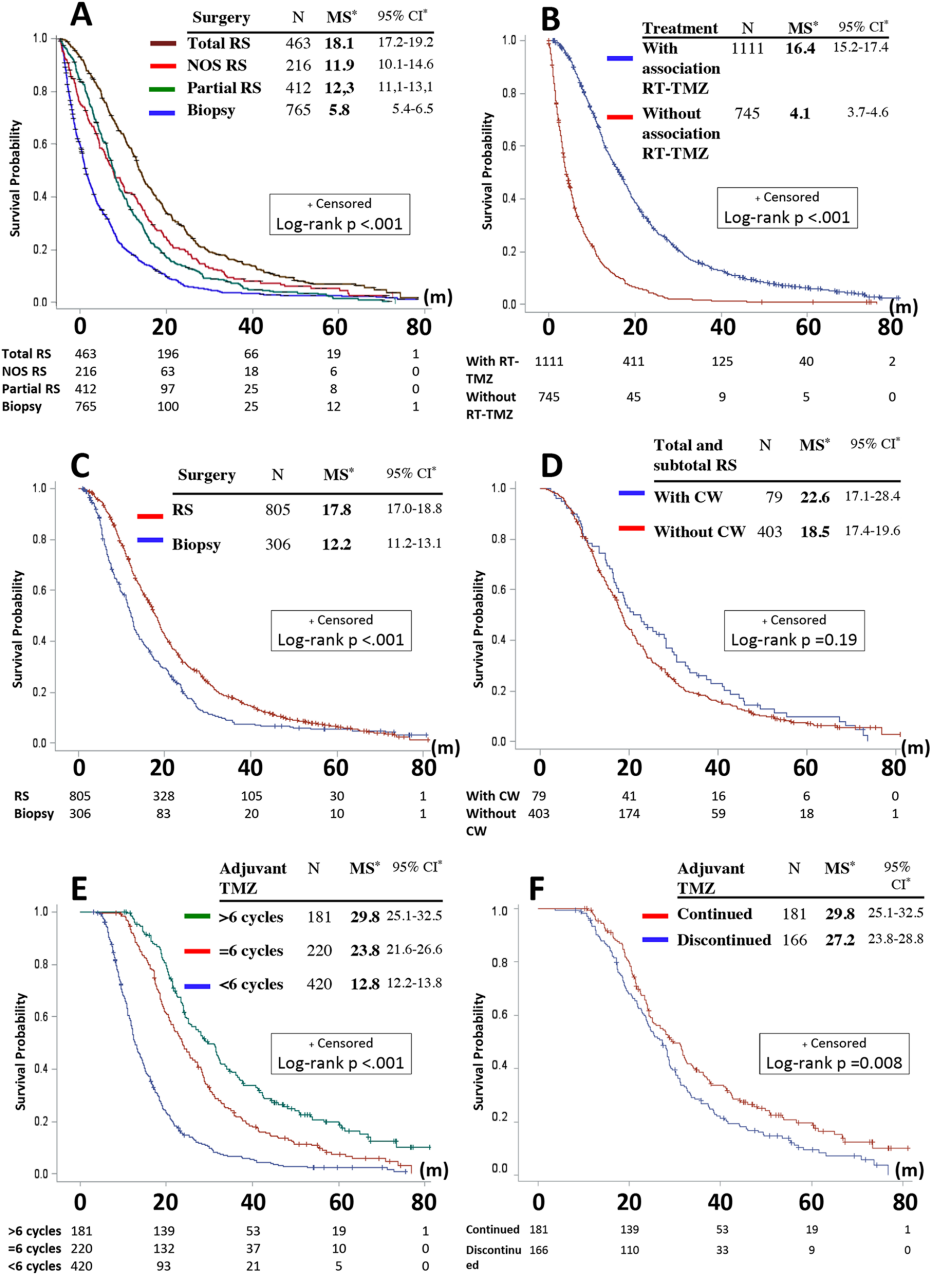
Survival and treatment patterns. Kaplan–Meier estimates of survival by: **a** first surgery (total RS, partial RS, NOS RS, and biopsy), **b** treatment including or excluding the combination of radiotherapy and temozolomide (RT–TMZ) in first-line treatment, **c** surgery (RS vs. biopsy) in the group of patients who initiated (at least) the RT–TMZ, **d** treatment with versus without local chemotherapy (carmustine wafer, CW) in the group of patients with total or subtotal RS and who initiated (at least) the RT–TMZ, **e** number of cycles of temozolomide (TMZ) (< 6, = 6, > 6c) in the group of patients who received adjuvant TMZ, **f** continuation versus discontinuation of the adjuvant TMZ after 6 cycles as defined by the Stupp protocol (in the group of patients free of progression). *Median survival (MS) and confidence interval (CI) are expressed in months (m). *c* Cycle, *NOS* not otherwise specified, *RS* resection

Overall, the two main therapeutic prognostic factors were extent of RS and combined RT–temozolomide treatment (Fig. 1a, b). Analysis of survival according to the first surgery (total RS, partial RS, not otherwise specified RS and biopsy) showed significant differences in MS, as follows: 18.1 (95% CI 17.2–19.2), 12.3 (95% CI 11.1–13.1), 11.9 (95% CI 10.1–14.6), and 5.8 (95% CI 5.4–6.5) months respectively, p < 0.001, log-rank test. Analysis of survival of group 1 versus 2 (according to first-line therapy including or excluding the combined RT–temozolomide treatment) showed significant differences in MS, as follows: MS for group 1 = 16.4 (95% CI 15.2–17.4) months and MS for group 2 = 4.1 (95% CI 3.7–4.6) months, p < 0.001, log-rank test. But, there were significant differences in patient characteristics between the groups. Patients in group 1 were significantly younger, with less median and bilateral lesion, with better KPS, with more RS versus biopsy, and with more total RS versus other RS than group 2 (Supplementary Table 1). After adjusting for age in quartiles and type of first surgery, mortality was higher for treatment without RT–temozolomide versus treatment with RT–temozolomide, HR = 2.8 (95% CI 2.5–3.1) (Supplementary Table 2). When KPS was introduced into the model as reported value (n = 861 patients) and/or as missing value (n = 1856 patients), results were very similar (Supplementary Table 3).

Survival in group 1 according to the surgery (RS, n = 805 vs. biopsy, n = 306) showed a significant difference: MS for group 1 with RS = 17.8 (95% CI 17.0–18.8) and MS for group 1 with biopsy = 12.2 (95% CI 11.2–13.1) months, p < 0.001, log-rank test (Fig. 1c).

Survival in the RS group (n = 805), according to the local CT, when analysis is limited to the patients with the information of total or subtotal RS (n = 482, 79 patients received CW and 403 did not receive CW), MS were 22.6 (95% CI 17.1–28.4) and 18.5 (95% CI 17.4–19.6) months, respectively, p = 0.19, log-rank test (Fig. 1d).

OS for the 821 patients who initiated the adjuvant phase was 18.9 (95% CI 18.0–19.8) months. Among them, 401 patients received 6 cycles or more of adjuvant temozolomide (MS = 25.5, 95% CI 24.0–28.3 months). According to the number of received cycles of adjuvant temozolomide (< 6 cycles: n = 420, = 6 cycles: n = 220, > 6 cycles: n = 181), MS showed significant difference and were: 12.8 (95% CI 12.2–13.8), 23.8 (95% CI 21.6–26.6), and 29.8 (95% CI 25.1–32.5) months, respectively, p < 0.001, log-rank test (Fig. 1e). Pairwise comparisons were statistically significant, p < 0.001 for all comparisons. Survival in the subgroups “<6, =6,>6 cycles”, according to the surgery (RS vs. biopsy) is shown in Supplementary Fig. S3.

Of the 220 patients who received strictly 6 cycles of adjuvant temozolomide, the treatment termination information for “end of treatment as defined by the standard treatment protocol” [6] (and not for progression or other reason) was specified in 166 cases. Analysis of survival based on discontinuation of the adjuvant temozolomide after the 6th cycle based on the definition of the protocol versus the continuation until progression showed significant differences in MS, as follows: 27.2 (95% CI 23.8–28.8) and 29.8 (95% CI 25.1–32.5) months, respectively, p = 0.008, log-rank test (Fig. 1f). These two subgroups were not significantly different for age or KPS, whereas the subgroup “continuation” (with the higher MS) received significantly less total RS (Table 3). After adjustment for surgery (total RS vs. other), the risk of mortality was significantly higher for patients who stopped at 6 cycles than those who continued the treatment, HR = 1.5 (95% CI 1.2–1.9) (Table 4).

**Table 3:** Comparison of main characteristics of patients receiving 6 cycles as defined by the protocol [sub-group 1, of the 220 patients who received strictly 6 cycles of adjuvant temozolomide, the treatment termination information for “end of treatment as defined by the protocol” (and not for progression or other reason) was specified in 166 cases] versus more than 6 cycles (sub-group 2) of adjuvant temozolomide

Characteristic (no. reported)	Sub-group 1 “discontinued” (N = 166) n (%)	Sub-group 2 “continued” (N = 181) n (%)	p
Age per quartile, in years (347)			0.61
≤ 56	65 (39.16)	83 (45.86)	
57–63	45 (27.11)	47 (25.97)	
64–72	39 (23.49)	36 (19.89)	
> 72	17 (10.24)	15 (8.29)	
Preoperative KPS (163)	(Missing data: 102)	(Missing data: 82)	0.92
90–100%	42 (65.63)	63 (63.64)	
70–80%	19 (29.69)	30 (30.30)	
≤ 60%	3 (4.69)	6 (6.06)	
First surgery (347)			< 0.001
“Total” RS	91 (54.82)	57 (31.49)	
Partial RS	38 (22.89)	57 (31.49)	
NOS RS	18 (10.84)	26 (14.36)	
Biopsy	19 (11.45)	41 (22.65)	
First surgery (347)			< 0.001
“Total” RS	91 (54.82)	57 (31.49)	
Other surgery	75 (45.18)	124 (68.51)	

*KPS* Karnofsky performance status, *NOS* not otherwise specified, *RS* resection

**Table 4:** Relative risk of mortality: uni- and multi-variate analysis for patients who discontinued temozolomide as defined by the protocol and for patients who continued temozolomide until progression

Variables	N	Nb (%) of deceased patients	Univariate analysis		Multivariate analysis	
			Hazard ratio (95% CI)	p values	Hazard ratio (95% CI)	p values
Treatment				0.009		0.001
“Continued” (> 6 cycles of adjuvant temozolomide)	181	139 (76.8)	1		1	
“Discontinued” (= 6 cycles of adjuvant temozolomide)	166	147 (88.6)	1.364 (1.082–1.721)		1.493 (1.172–1.903)	
Age (years)				0.07		Excluded from model (NS)
≤ 56	148	120 (81.1)	1			
57–63	92	72 (78.3)	1.051 (0.785–1.408)			
64–72	75	64 (85.3)	1.391 (1.026–1.884)			
> 72	32	30 (93.8)	1.485 (0.994–2.219)			
Surgery				0.10		0.01
Total resection	148	119 (80.4)	1		1	
Other surgery	199	167 (83.9)	1.218 (0.963–1.542)		1.368 (1.070–1.749)	

MS in group 2 (n = 745) was 4.1 (95% CI 3.7–4.6) (Fig. 1b) and 5.9 (95% CI 5.5–6.6) months when patients with biopsy only were excluded. Main subgroup MS (months) were: 1.8 (95% CI 1.6–2.1) for biopsy only, 5.3 (95% CI 4.8–6.5) for biopsy + CT, 7.2 (95% CI 5.2–8.7) for biopsy + RT, 6.8 (95% CI 5.9–9.1) for RS + CT, and 10.8 (95% CI 9.4–12.9) for RS + RT.

## Discussion

This study, including all patients with newly-diagnosed and histologically confirmed GBM, detailed the proportions of patients who: (1) initiated the standard treatment, (2) initiated the adjuvant phase, (3) received strictly 6 or > 6 cycles of adjuvant TMZ before progression. MS were estimated for each group, and we showed that prolonging the adjuvant temozolomide beyond 6 cycles positively impacted survival in non-progressive patients.

In our study, the crude incidence rate was 3.3/100,000 person-years and is in good accordance with the literature [1]. OS was 11.2 months, similar to the values obtained in population-based studies in the post-temozolomide era [28–30]. Survival rates were similar to US results [2], and intermediate between those of the RT-only arm and those of the RT–temozolomide arm in the EORTC–NCIC clinical trial [31]. Woehrer et al. reviewed GBM survival in different population studies and showed a significant but modest survival gain over time [17]. We can explain this modest survival gain: (i) in “real world”, only 60% of all patients initiate the standard treatment, (ii) less than a quarter of all patients received 6 cycles or more of adjuvant temozolomide, and (iii) elderly patients have been treated more often in recent years.

Some previous papers have summarized the first-line treatment in population studies. For example, after surgery in the works of Graus et al. [32], Brandes et al. [33], and in our series, 57%/62.5%/59.9% of all patients received the combined RT–temozolomide treatment, 21%/15.7%/20.1% received other regimens, and 22%/21.7%/20% were not treated, respectively. But, to our knowledge, only one recent paper described the detail and outcome of the different phases of the standard protocol in a large population-based study, and according to the surgery [30]. The results from the Danish population-based study and our results (main spontaneous prognostic factors, OS, survival according to the surgery and to postoperative treatment, etc.) were very close. This highlights the interest of nationwide population studies.

### Survival according to the duration of maintenance therapy of temozolomide

The question of the optimal duration of maintenance therapy of temozolomide remains controversial. Some institutions stop the adjuvant temozolomide after 6 cycles in accordance with the protocol, whilst others prolong treatment up to 12 or even more cycles in non-progressive patients. In daily practice, the prescribed number of cycles for patients without tumor progression after 6 months varies greatly. Many previous and ongoing trials of CT for malignant gliomas prescribe maintenance temozolomide for up to 12 months [34–36]. Early publications on this subject claimed prolonged survival of patients receiving temozolomide treatment extended beyond 6 cycles [37–40]. In contrast, three recent interesting works concluded that continuing temozolomide beyond 6 cycles does not show longer OS [35, 41, 42]. But none of them was a specific clinical trial or a real population-based study. Blumenthal et al. [35] performed an interesting pooled analysis of individual patient data from four randomized trials for newly diagnosed GBM. A total of 2214 GBM patients were included in the four trials. All patients who were progression free 28 days after cycle 6 were included. The decision to continue temozolomide was per local practice and standards, and at the discretion of the treating physician. Of these, 624 qualified for analysis: 291 continued maintenance temozolomide until progression or up to 12 cycles, while 333 discontinued temozolomide after 6 cycles (these nearly equivalent numbers illustrate the difficulty of the question). First, as noted by the authors, this study is an unplanned retrospective meta-analysis of patients included in clinical trials spanning a decade. Secondly, these patients were selected and were not representative of the population in the real world (age, percentage of complete RS, comorbidity, etc.). Thirdly, whilst the percentage of approximately 30% of patients remained progression free at the end of the standard 6 cycles of temozolomide maintenance therapy is in accordance with data from clinical trials, it is high compared with data from population-based or real world studies. This could reflect the frequent selectivity of patients in inclusion in clinical trials. Fourthly, the pattern of patients free of progression after the 6th adjuvant temozolomide, includes in these randomized studies was different than ours, particularly in proportion of total RS (53.7 vs. 42.7%). This better prognostic factor is less frequent in real life population patients and participates to increase the heterogeneity [30]. We can speculate that prolonging temozolomide beyond 6 cycles could offer benefit in OS to a subset of patients that could not have been operated in a maximal approach (for different reason: age, comorbidity, tumor topography, etc.). But unfortunately, our work does not allow us to identify which subgroup of patients benefit most from the continuation of treatment.

### Note concerning the French oncological management for glioblastoma

There is not typical peculiarity. However, firstly, we can notice that 6.2% of the patients only, were operated in private neurosurgical centers while French hospitals (in general) include 33% of private centers (Direction générale de l’offre de soins, DGOS, https://solidarites-sante.gouv.fr/IMG/pdf/dgos_cc_2018_02_16_a_web_pages_hd.pdf). This difference can be explained by the fact that there is a very strict regulation for the brain tumors management in France. Secondly, here, the percentage of RS (59%) is lower than in many studies. But most data come from clinical trials or studies from specific centers, while accurate neurosurgical data from population-based studies are limited. Thirdly, the management (median dose 60 Gy) and the percentage of patients who received RT as first-line treatment (68%) were similar as in many countries. Fourthly, concerning the duration of maintenance therapy of temozolomide before recurrence, no recommendation does exist in France. Some centers treated with 6 cycles strictly while other treated during 1 year, or even longer.

### Limitations

Our study did not include any biological data [i.e., O_6_–methylguanine DNA methyltransferase (MGMT) promoter methylation status, or isocitrate dehydrogenase (IDH) mutation status], nor the presence of residual tumor after the 6th cycle (e.g., our study did not provide information on the reasons why the temozolomide was continued, such as the persistence (or not) of enhanced lesions on imaging, or the knowledge of the MGMT status). Therefore, the main limitation of our study is that we cannot formally eliminate the existence of a bias in the result of the survival analysis according to the duration of maintenance therapy of temozolomide.

However, for the comparison of the survival between the two groups (patients who received strictly 6 cycles of adjuvant temozolomide vs. patients who received > 6 cycles), we included only patients with the information of “end of treatment as defined by the standard treatment protocol” and not for progression or other reason, in the group with strictly 6 cycles. Moreover, these two groups were not significantly different for age or KPS, whereas the group “continuation” (with the higher MS) received significantly less total RS (Table 3). The multivariate model showed that the risk of mortality was significantly higher for patients who stopped at 6 cycles than those who continued the treatment (Table 4). Unfortunately, among the patients who continued the treatment after 6 cycles, we can not identify which subgroup of patients benefited most from continuation of treatment despite methodological efforts. One of the main hypothesis could be low activity of MGMT via methylation of its promoter. And, we can hypothesize that persistence of an enhanced lesion on MRI after 6 cycles, and/or positive methylated status, might be one of the best indication.

### Perspective

Few countries have national registry for brain tumors (e.g., USA, Scandinavian countries, Austria, etc.). Sometimes they participate in international works about epidemiology, biology, or genetic analysis, but to our knowledge international population-based study analyzing oncological management for the entire population of several countries does not exist. International population-based studies analyzing oncological management for brain tumors (e.g., GBM) would be an opportunity to answer clinical questions.

## Conclusions

To our knowledge, this is the first work detailing the first-line treatment including the duration of maintenance therapy of temozolomide, and survival in a large GBM patient population-based study. We showed that prolonging the adjuvant temozolomide beyond 6 cycles in non-progressive patient positively impacted survival. Complementary analysis including adverse effects of the treatments, quality of life, and biology, warrant a dedicated randomized clinical trial, or a large prospective international population-based study.

## Electronic supplementary material

Below is the link to the electronic supplementary material.

Supplementary material 1 (DOCX 426 KB)
