# Supplementary material for: Association of patterns of care, prognostic factors, and use of radiotherapy–temozolomide therapy with survival in patients with newly diagnosed glioblastoma: a French national population-based study
Source: J Neurooncol. 2018 Dec 6;142(1):91–101. doi: 10.1007/s11060-018-03065-z (PMC6399437; doi:10.1007/s11060-018-03065-z)
Supplement: Supplementary file 1 — Supplementary material 1 (DOCX 426 KB) [file 11060_2018_3065_MOESM1_ESM.docx]

**Supplementary Material**

**Supplementary Table 1** Main differences in the clinical and surgical characteristics of group 1 (first-line including combined radiotherapy and temozolomide treatment) and group 2 (first-line excluding combined radiotherapy and temozolomide treatment)

**Supplementary Table 2** Relative risk of mortality: multivariate Cox analysis with treatment including or excluding combined radiotherapy and temozolomide treatment, age at diagnosis, and modality of the first surgery

**Supplementary Table 3** Relative risk of mortality: multivariate Cox analysis of the population including the knowledge (or not) of Karnofsky performance status

**Supplementary Figure S1** Overall survival and survival rates

**Supplementary Figure S2** Survival and spontaneous prognostic factors: A, Survival and age; B, Survival and location; C, Survival and Karnofsky performance status; D, Survival in reported and unreported Karnofsky performance status groups

**Supplementary Figure S3** Impact of the surgery (resection vs. biopsy) in the group of patients who received adjuvant temozolomide, according to the number of received cycles (c) of temozolomide (<6c: A), (=6c: B), and (>6c: C)

**Supplementary Table 1** Main differences in the clinical and surgical characteristics of group 1 (first-line including combined radiotherapy and temozolomide treatment) and group 2 (first-line excluding combined radiotherapy and temozolomide treatment)

|  | **Group 1 (N=1111)**  **n (%)** | **Group 2**  **(N=745)**  **n (%)** | **p** |
| --- | --- | --- | --- |
| Age per quartile (no. reported: 1856) |  |  | <0.001 |
| ≤56 years | 357 (32.13) | 112 (15.03) |  |
| 57-63 years | 312 (28.08) | 110 (14.77) |  |
| 64-72 years | 303 (27.27) | 184 (24.70) |  |
| >72 years | 139 (12.51) | 339 (45.50) |  |
| Location of the tumor (no. reported: 1734) |  |  | <0.001 |
| Right | 521 (48.56) | 298 (45.08) |  |
| Left | 504 (46.97) | 289 (43.72) |  |
| Median and/or bilateral | 48 (4.47) | 74 (11.2) |  |
| Preoperative KPS (no. reported: 861) |  |  | <0.001 |
| 90%-100% | 299 (58.86) | 128 (36.26) |  |
| 70%-80% | 171 (33.66) | 149 (42.21) |  |
| ≤60% | 38 (7.48) | 76 (21.53) |  |
| First surgery (no. reported: 1856) |  |  | <0.001 |
| “Total” RS | 383 (34.47) | 80 (10.74) |  |
| Partial RS | 292 (26.28) | 120 (16.11) |  |
| NOS RS | 130 (11.70) | 86 (11.54) |  |
| Biopsy | 306 (27.54) | 459 (61.61) |  |

Abbreviations: KPS, Karnofsky performance status; NOS, not otherwise specified; RS, resection

**Supplementary Table 2** Relative risk of mortality: multivariate Cox analysis with treatment including or excluding combined radiotherapy and temozolomide treatment, age at diagnosis, and modality of the first surgery

| **Variable** | **n** | **Hazard Ratio (95% CI)** | ***P* Value** |
| --- | --- | --- | --- |
| **Treatment** |  |  | <.001 |
| Including RT-temozolomide | 1111 | 1 |  |
| Excluding RT-temozolomide | 745 | 2.815 (2.516-3.149) |  |
| **Age** |  |  | <.001 |
| ≤56 y | 469 | 1 |  |
| 57-63 y | 422 | 1.248 (1.086-1.434) | 0.002 |
| 64-72 y | 487 | 1.448 (1.266-1.657) | <.001 |
| >72 y | 478 | 1.477 (1.280-1.705) | <.001 |
| **Surgery** |  |  | <.001 |
| “Total” RS | 463 | 1 |  |
| Partial RS | 412 | 1.409 (1.225-1.619) | <.001 |
| Not otherwise specified RS | 216 | 1.163 (0.980-1.381) | 0.08 |
| Biopsy | 765 | 1.721 (1.514-1.956) | <.001 |

Abbreviations: 95% CI, 95% Confidence Interval; RS, resection; RT, radiotherapy; RT-temozolomide, combined radiotherapy and temozolomide treatment

**Supplementary Table 3** Relative risk of mortality: multivariate Cox analysis of the population including the knowledge (or not) of Karnofsky performance status

| **Variable** | **Factor** | **Multivariate Analysis with reported KPS**  **(N= 861)** | | | | **Multivariate Analysis with reported KPS and as missing data (N=1856)** | | | |
| --- | --- | --- | --- | --- | --- | --- | --- | --- | --- |
|  |  | n | Hazard Ratio | | p Value | n | Hazard Ratio | | p Value |
|  |  |  | Estimate | 95% CI |  |  | Estimate | 95% CI |  |
|  |  |  |  |  |  |  |  |  |  |
| **Treatment** | With RT-temozolomide | 508 | 1 |  |  | 1111 | 1 |  |  |
|  | Without RT-temozolomide | 353 | 2.689 | 2.275 - 3.178 | **<.0001** | 745 | 2.770 | 2.474 - 3.102 | **<.0001** |
| **Surgery** |  |  |  |  | **<0.001** |  |  |  | **<0.001** |
|  | “Total” RS | 220 | 1 |  |  | 463 | 1 |  |  |
|  | Partial RS | 219 | 1.302 | 1.066 - 1.591 | **0.0097** | 412 | 1.372 | 1.192 - 1.579 | **<.0001** |
|  | NOS RS | 85 | 1.157 | 0.881 - 1.520 | 0.2920 | 216 | 1.139 | 0.959 - 1.353 | 0.1371 |
|  | Biopsy | 337 | 1.670 | 1.382 - 2.019 | **<.0001** | 765 | 1.709 | 1.503 - 1.943 | **<.0001** |
| **Age** |  |  |  |  | **0.0034** |  |  |  | **<0.0001** |
|  | ≤56 y | 216 | 1 |  |  | 469 | 1 |  |  |
|  | 57-63 y | 191 | 1.134 | 0.920 - 1.397 | 0.2384 | 422 | 1.250 | 1.088 - 1.436 | **0.0017** |
|  | 64-72 y | 227 | 1.334 | 1.090 - 1.632 | **0.0051** | 487 | 1.420 | 1.240 - 1.625 | **<.0001** |
|  | >72 y | 227 | 1.454 | 1.170 - 1.806 | **0.0007** | 478 | 1.444 | 1.250 - 1.668 | **<.0001** |
| **KPS** |  |  |  |  | **0.0004** |  |  |  | **0.0011** |
|  | 90%-100% | 427 | 1 |  |  | 427 | 1 |  |  |
|  | 70%-80% | 320 | 1.261 | 1.076 - 1.477 | **0.0041** | 320 | 1.234 | 1.057 - 1.441 | **0.0077** |
|  | ≤60% | 114 | 1.527 | 1.214 - 1.920 | **0.0003** | 114 | 1.480 | 1.186 - 1.848 | **0.0005** |
|  | MD | - | - | - | - | 995 | 1.209 | 1.072 - 1.363 | **0.0020** |

Abbreviations: 95% CI, 95% Confidence Interval; KPS, Karnofsky performance status, MD, missing data; NOS, not otherwise specified; RS, resection; RT, radiotherapy; RT-temozolomide, combined radiotherapy-temozolomide treatment

**Supplementary Figure S1** Overall survival and survival rates


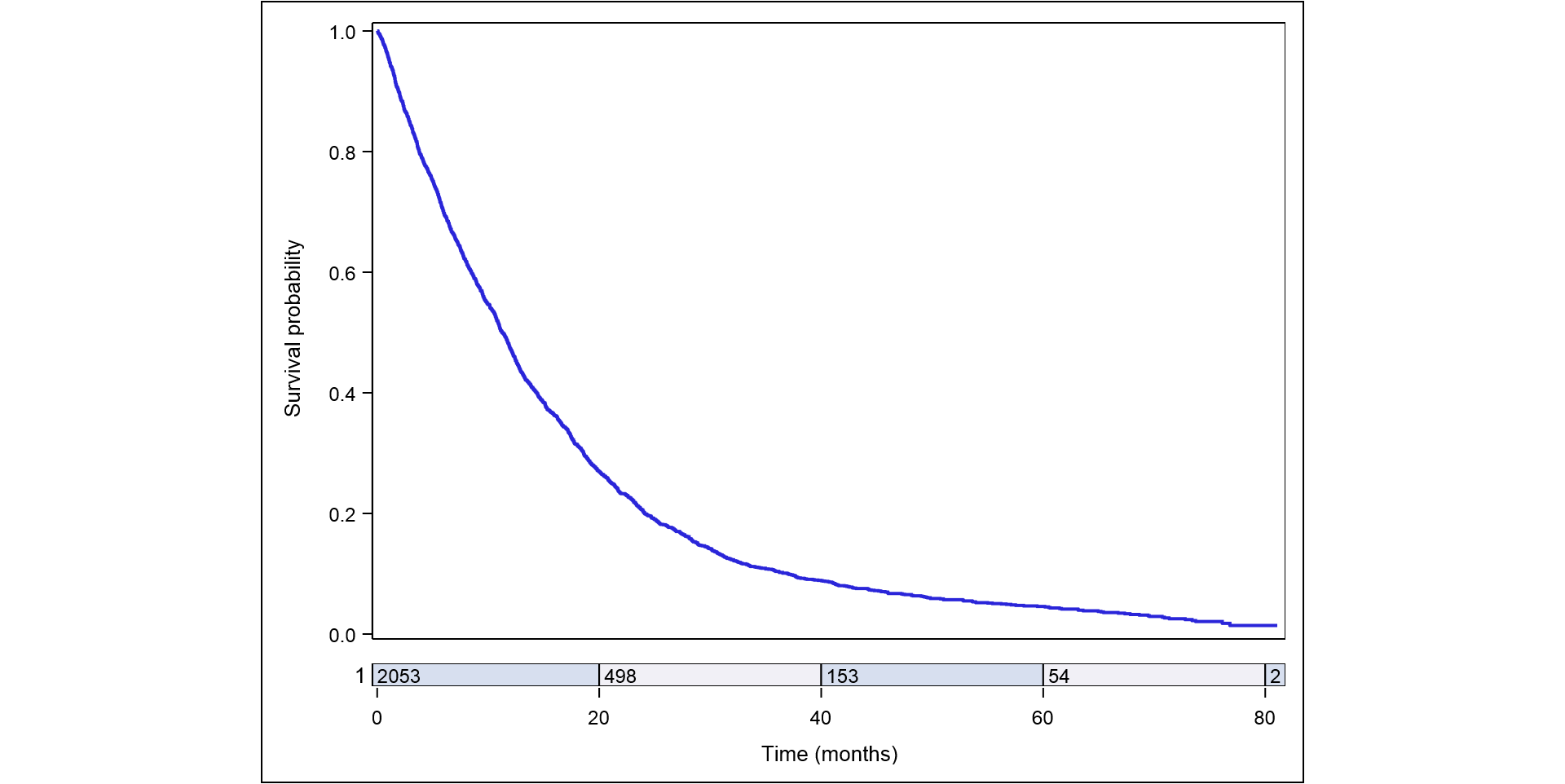


The numbers just below the x-axis represent the numbers at risk.

Median overall survival: 11.2 (95% CI, 10.7-11.9) months

**Survival rates**

|  | **1-year** | **1.5-year** | **2-year** | **3-year** | **4-year** | **5-year** |
| --- | --- | --- | --- | --- | --- | --- |
| **Survival rate (%)**  **95% CI (%)** | 47.1  44.8-49.3 | 31.4  29.3-33.5 | 20.1  18.3-22.0 | 10.3  9.0-11.8 | 6.4  5.3-7.6 | 4.5  3.6-5.6 |

**Supplementary Figure S2A** Survival and spontaneous prognostic factors: Survival and age (per quartile)

**Log-rank p <0.001**


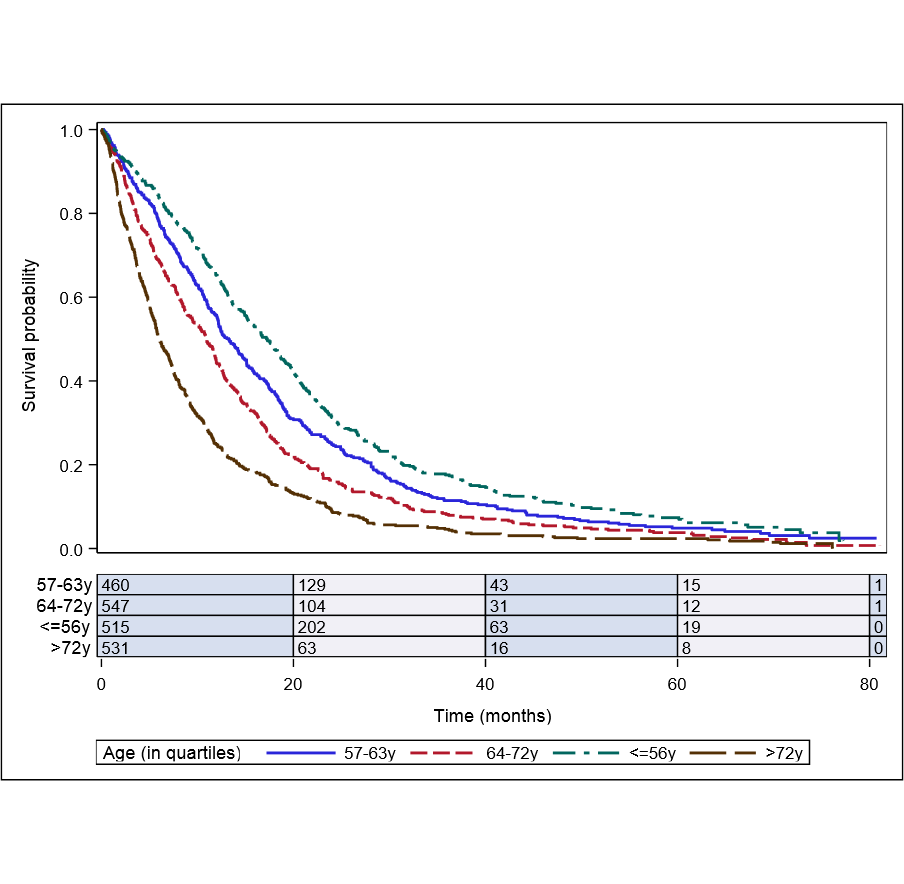


The numbers just below the x-axis represent the numbers at risk

| Age (years) | n | Median survival (95% Confidence Interval) months |
| --- | --- | --- |
| <=56 | 515 | 17.2 (15.3-18.7) |
| 57-63 | 460 | 13.2 (12.1-14.9) |
| 64-72 | 547 | 10.9 (9.5-11.8) |
| >72 | 531 | 5.9 (5.5-7.0) |

**Supplementary Figure S2B** Survival and spontaneous prognostic factors: Survival and location (right, left, median and bilateral, and unknown)

**Log-rank p <0.001**


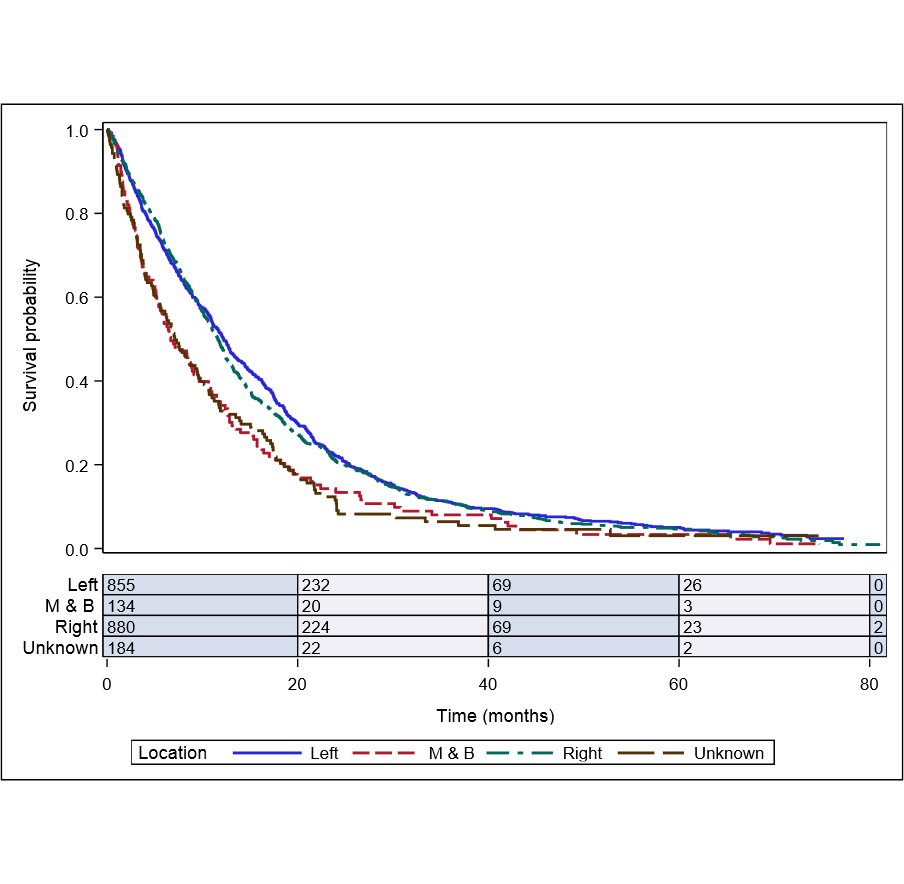


The numbers just below the x-axis represent the numbers at risk

Abbreviation: M&B, median and bilateral

| Location | n | Median survival (95% Confidence Interval) months |
| --- | --- | --- |
| M&B | 134 | 6.7 (5.4-9.1) |
| Right | 880 | 11.6 (10.8-12.3) |
| Left | 855 | 12.2 (11.1-13.0) |
| Unknown | 184 | 7.3 (5.5-9.5) |

**Supplementary Figure S2C** Survival and spontaneous prognostic factors: Survival and Karnofsky performance status

**Log-rank p <0.001**


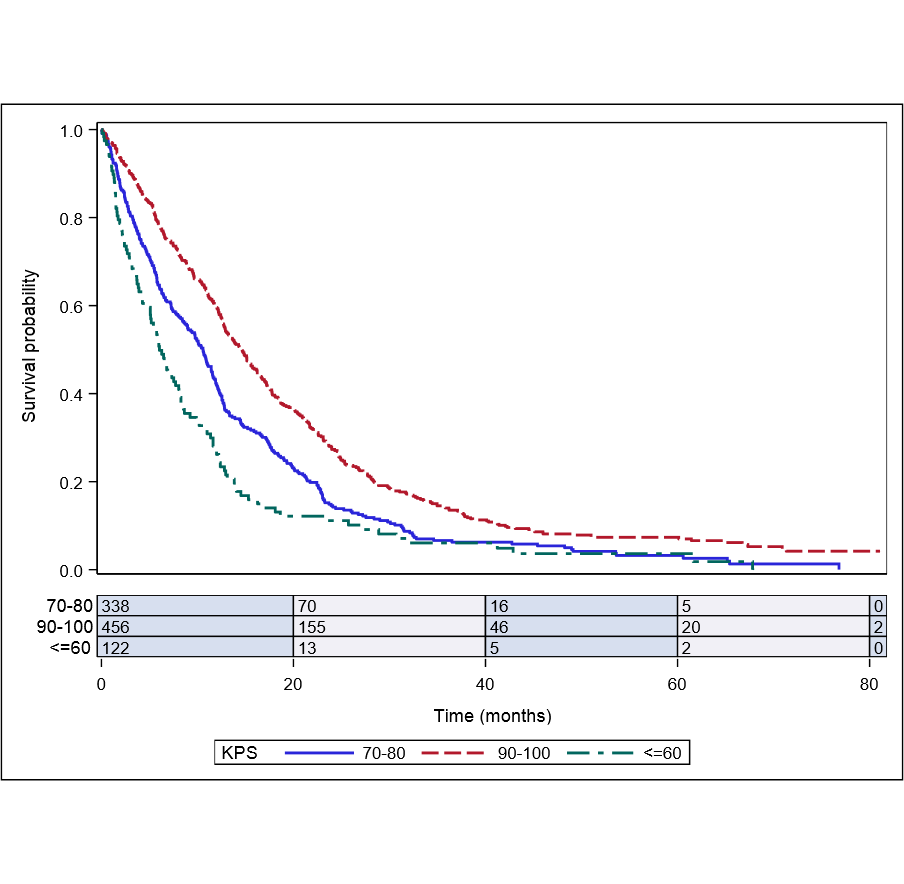


The numbers just below the x-axis represent the numbers at risk

Abbreviation: KPS, Karnofsky performance status

| KPS | n | Median survival (95% Confidence Interval) months |
| --- | --- | --- |
| 90%-100% | 456 | 14.5 (12.8-15.9) |
| 70%-80% | 338 | 10.6 (8.9-11.6) |
| <=60 | 122 | 6.0 (5.1-8.0) |

**Supplementary Figure S2D** Survival and spontaneous prognostic factors: Survival in reported and unreported Karnofsky performance status groups

**Log-rank p =0.50**


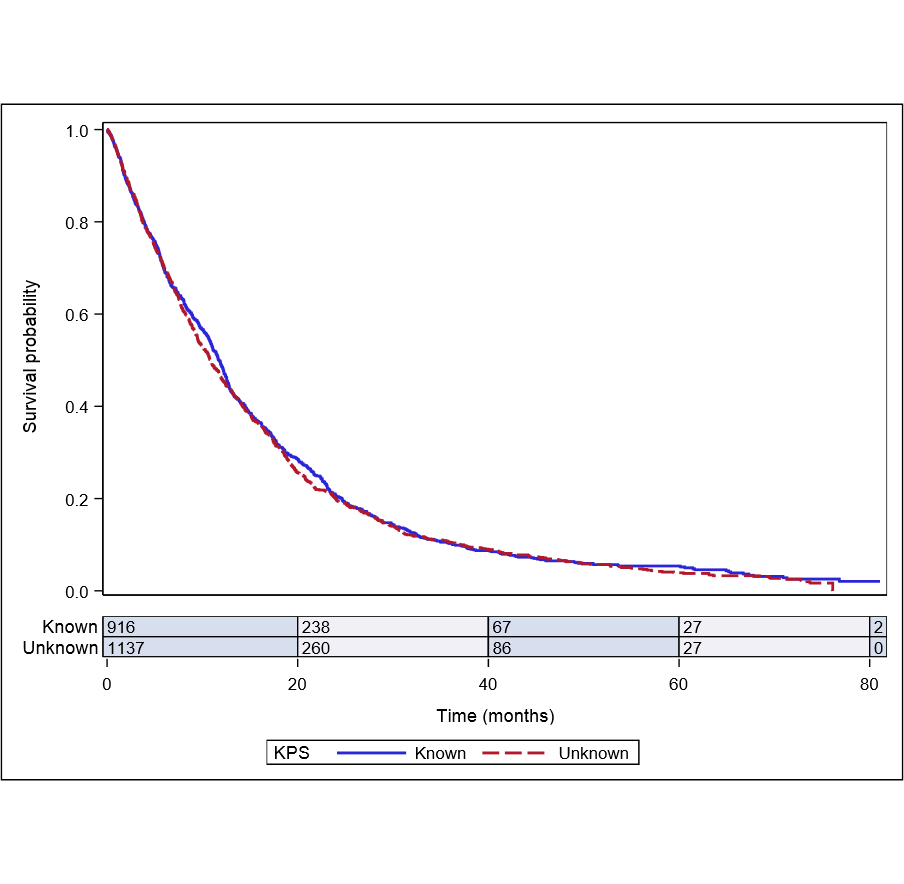


The numbers just below the x-axis represent the numbers at risk

Abbreviation: KPS, Karnofsky performance status

| KPS | n | Median survival (95% Confidence Interval) months |
| --- | --- | --- |
| Known | 916 | 11.7 (10.9-12.4) |
| Unknown | 1137 | 10.9 (10.0-11.8) |

**Supplementary Figure S3A** Impact of the surgery (resection vs. biopsy) in the group of patients who received adjuvant temozolomide, according to the number of received cycles (c) of TMZ (<6c)

**Log-rank p =0.01**


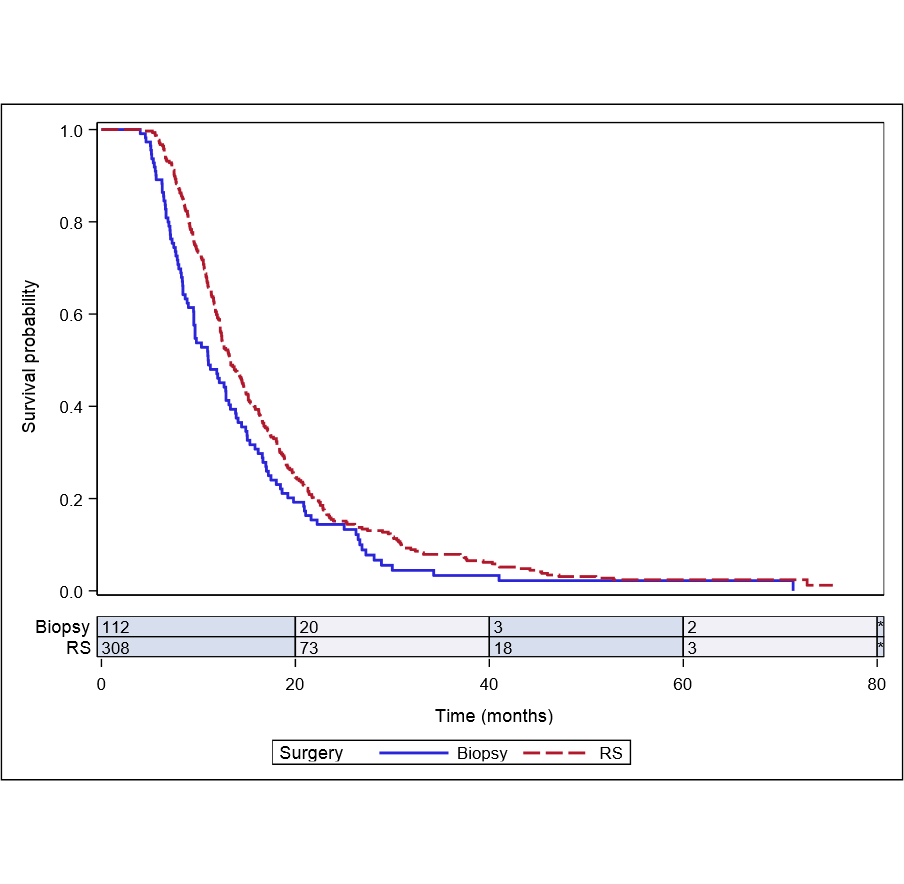


The numbers just below the x-axis represent the numbers at risk

Abbreviation: RS, resection

| Surgery | n | Median survival (95% Confidence Interval) months |
| --- | --- | --- |
| Resection | 308 | 13.3 (12.4-14.7) |
| Biopsy | 112 | 11.0 (9.5-13.1) |

**Supplementary Figure S3B** Impact of the surgery (resection vs. biopsy) in the group of patients who received adjuvant temozolomide, according to the number of received cycles (c) of TMZ (=6c)

**Log-rank p =0.02**


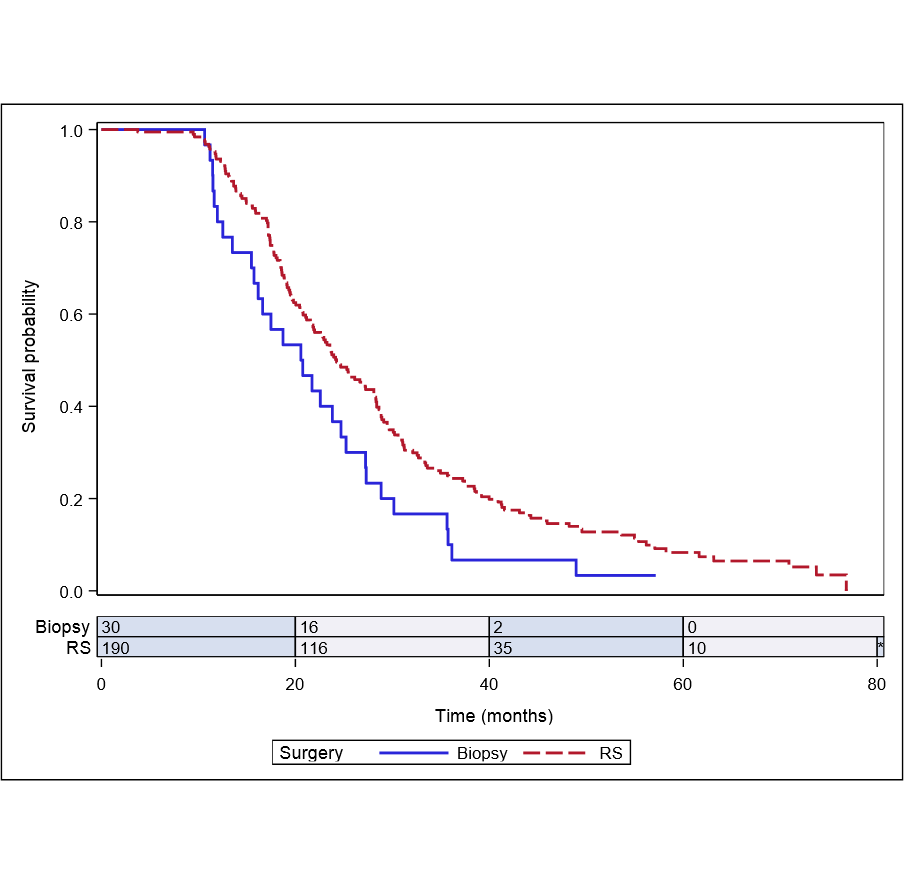


The numbers just below the x-axis represent the numbers at risk

Abbreviation: RS, resection

| Surgery | n | Median survival (95% Confidence Interval) months |
| --- | --- | --- |
| Resection | 190 | 24.2 (21.8-28.1) |
| Biopsy | 30 | 20.7 (15.7-25.2) |

**Supplementary Figure S3C** Impact of the surgery (resection vs. biopsy) in the group of patients who received adjuvant temozolomide, according to the number of received cycles (c) of TMZ (>6c)

**Log-rank p: NA**


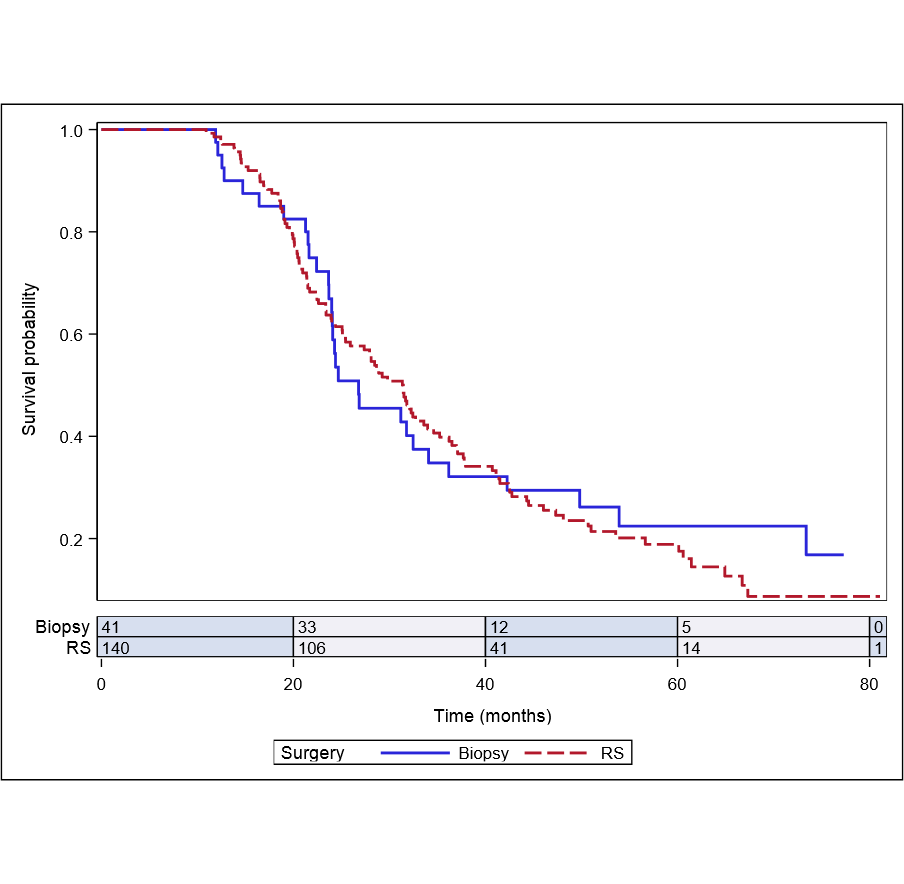


The numbers just below the x-axis represent the numbers at risk

Abbreviation: RS, resection

| Surgery | n | Median survival (95% Confidence Interval) months |
| --- | --- | --- |
| Resection | 140 | 31.4 (25.4-34.0) |
| Biopsy | 41 | 26.8 (23.7-34.1) |
